# Impact of an organization-wide knowledge translation strategy to support evidence-informed public health decision making

**DOI:** 10.1186/s12889-018-6317-5

**Published:** 2018-12-29

**Authors:** Maureen Dobbins, Robyn L. Traynor, Stephanie Workentine, Reza Yousefi-Nooraie, Jennifer Yost

**Affiliations:** 1 School of Nursing, Faculty of Health Sciences, McMaster University, 175 Longwood Road, South Suite 210A, Hamilton, Ontario Canada; 2 Department of Community Health & Epidemiology, Dalhousie University, Halifax, Nova Scotia Canada; 3 Alberta Health Services, 10101 Southport Road SW, Calgary, AB T2W 3N2 Canada; 4 Department of Public Health Sciences, University of Rochester, Rochester, New York, USA; 5 M. Louise Fitzpatrick College of Nursing, Villanova University, Driscoll Hall, Room 330, 800 Lancaster Avenue, Villanova, PA USA

**Keywords:** Evidence-informed decision making, Knowledge translation, Knowledge broker, Public health, Diffusion of innovations, Organizational change

## Abstract

**Background:**

The public health sector is moving toward adopting evidence-informed decision making into practice, but effort is still required to effectively develop capacity and promote contextual factors that advance and sustain it. This paper describes the impact of an organization-wide knowledge translation intervention delivered by knowledge brokers on evidence-informed decision making knowledge, skills and behaviour.

**Methods:**

A case study design was implemented with the intervention and data collection tailored to the unique needs of each case (health department). A knowledge broker provided training workshops and mentored small groups through a seven step process of evidence-informed decision making. The intervention was delivered over 22 months; data related to evidence-informed decision making knowledge, skills and behaviour were collected at baseline and follow-up. Mixed effects regression models were developed to assess the impact of involvement in the intervention on the evidence-informed decision making outcomes.

**Results:**

Data from a total of 606 health department staff were collected during baseline: 207 (33%) staff from Case A, 304 (28%) from Case B, and 95 (47%) from Case C. There were a total of 804 participants at follow-up: 258 (42%) from Case A, 391 from Case B (37%), and 155 (50%) from Case C. Statistically significant increases in knowledge and skills were observed overall, and in all three health departments. An increase in evidence-informed decision making behaviour was observed among those intensively involved in the intervention from all cases (statistically significant in Case A). The organizational characteristics of strategic priority, leadership, readiness, and choice of staff emerged as important factors in the change process.

**Conclusions:**

Knowledge brokering is a promising organizational knowledge translation intervention to support evidence-informed decision making. The intervention appeared to have the greatest impact on those who became actively engaged with the knowledge broker in the intervention. Active participation in face-to-face training activities with a knowledge broker, focused specifically on evidence-informed decision making skill development, led to the greatest impact on associated behaviours, knowledge, and skills. Several organizational factors emerged as integral to success of the knowledge translation intervention.

## Background

Policy makers and the Canadian public have come to expect that the best available research evidence be used to inform public health practice and policy [1]. Evidence-informed decision making (EIDM) in public health involves considering the best available research evidence in combination with other factors such as available resources, client preferences, political climate, and health practitioner expertise [2–4]. Core competencies for public health practice, developed by the Public Health Agency of Canada, identify specific knowledge and skills related to EIDM that public health professionals should possess [5]. In addition, multiple provinces have standards in place for public health practice which stipulate that public health professionals should engage in EIDM [6–8]. While the expectation to engage in and demonstrate EIDM exists, public health professionals and organizations require support to acquire the necessary knowledge, skills, infrastructure, and context for EIDM.

Implementing and achieving EIDM in complex public health contexts has many challenges [1, 9–11]. Key challenges include: limited time; demanding workloads; competing priorities; emerging crises; limited capacity for searching, appraising, and applying research evidence; limited knowledge management skills and infrastructure; resistance to change; unsupportive organizational culture and leadership; the political context of decision making; and the ever-expanding evidence base [2, 11–17]. With many identified barriers to EIDM, there is a need to identify effective and sustainable knowledge translation (KT) strategies to enhance the capacity for public health organizations and workforce to operate in an evidence-informed way [2, 12, 14, 18].

The literature consistently suggests that active KT strategies are the most effective interventions, especially when customized to suit varying contexts [3, 13, 15, 19, 20]. KT is the science of applying research knowledge to decisions for healthcare services, policies, and programs, in an attempt to close the gap between research and practice [16, 21, 22]. Tailored and targeted messaging, knowledge management infrastructure, and multidirectional interaction between research and decision-making communities, in particular, show promise as effective KT strategies [3, 11, 13, 15]. Research indicates that strategies to support EIDM need to focus not only on building individual knowledge and skills, but also on shifting the culture within organizations to value EIDM and to develop infrastructure and mechanisms that support it [2, 12, 23]. In the last decade, knowledge brokers (KB)s have been shown to play a key role in facilitating and supporting EIDM in the public health sector [18, 24–26]. The role of a KB is to facilitate the process of moving evidence into action [27, 28]. KBs often act as a link or bridge between research producers and research users, promoting collaboration and interaction, and supporting the application of evidence in decision-making [24, 28].

The Health Evidence research team, in partnership with three Canadian public health departments, conducted a case study funded by the Canadian Institutes of Health Research (CIHR), Partnerships for Health System Improvement (PHSI) initiative (FRN 101867). A key component of this program was the collaborative relationship between researchers and public health decision-makers, with both parties serving as co-primary investigators on the study. The purpose of this study was to enhance individual capacity for and facilitate organizational contexts conducive to EIDM. We developed a KT intervention tailored to the needs of each participating health department and implemented by KBs. The objectives of this study were to: observe and describe the impact of a KT intervention on EIDM knowledge, skills, and behaviour; and identify contextual factors which facilitated or impeded impact.

## Methods

### Design

We used case study methodology with embedded units of analysis (organization). Case study involves the description, exploration and understanding of a contemporary phenomenon within its real-life context [29]. It is a particularly useful method of investigation when the phenomenon of interest involves complex social interactions, when investigators have minimal control over variables, and when boundaries between the phenomenon under study (the KT intervention) and the context in which it is situated (the health departments) are not clearly delineated [29]. A case study approach was considered to be the most rigorous and appropriate design for this study, because the intent was to observe and describe the impact of an organization-wide knowledge translation intervention as it naturally evolved, within each case. The purpose of the study therefore was less to do about evaluating the effectiveness of the KT intervention, and more about comparing how the intervention evolved across cases and whether and how individuals and the organizations changed over time. Furthermore, given public health departments vary considerably with respect to demographic, contextual and other factors, it was not possible to identify a control group that would be sufficiently similar to the intervention group cases to act as a comparator. A protocol with pre-defined assessment and procedure criteria was developed as part of the funding proposal submitted for review at the Canadian Institutes for Health Research (CIHR). The procedures identified in the proposal were implemented and reported on in this paper. Ethics approval for this project was granted by the McMaster University Faculty of Health Sciences Research Ethics Board.

### Study sample

The sample consisted of public health professionals from three public health departments in Ontario, with each department representing a unique case. The cases represented a mix of sizes and complexity, urbanization levels, committed resources and plans for EIDM, and proximity to the research team. Members of the research team had varying levels of experience working in public health prior to the study. Each case was within 125 km of the research team, which allowed for greater face-to-face interaction prior to and during the study. Public health professionals from each health department were actively involved in the development of the research proposal and throughout the study.

A senior manager was identified as a key contact from each health department. As a link between the research team and the health department, the key contact’s role was to assist in finalizing the KT intervention (i.e. how often the KB was on site, content of training), and facilitate implementation of the KT intervention and data collection by assessing study progress, addressing concerns, brainstorming new ideas, and identifying staff in the health department who could actively participate in the intervention. Participants from each health department included specialists (e.g. epidemiologists, consultants, Research and Policy Analysts (RPAs), dieticians, and nutritionists), management (e.g. directors, Associate Medical Officers of Health, Medical Officers of Health), and front-line practitioners (e.g. Public Health Nurses, Health Promotion Officers, Public Health Inspectors, and dental professionals). Informed written consent was obtained from all participants.

Differences in how the health departments were organized and their previous involvement in EIDM resulted in each using a different approach for involving staff in the intervention. Case A used a cohort approach in which 4–10 teams, consisting of 2–4 staff each, worked with the KB for a set period, forming a cohort. When the first cohort completed their work, a new cohort of teams was selected and began working with the KB. In Case C, five teams were selected at the outset of the study. These five teams worked with the KB throughout the intervention. Finally, Case B used a hybrid of these two approaches: staff were selected at the beginning of the study to be involved but their interaction with the KB was staggered over the course of the intervention.

### Knowledge translation intervention

The KT intervention was implemented in each case over 22 months with a staggered start date. Given the time required to deliver the KT intervention, it was necessary to have two KBs, where one KB was assigned two of the cases, and the other KB one case. Both KBs were Masters prepared, with one having a Masters of Science degree and the other a course-based Masters in Health Administration. Neither held a health professional designation. Both had several years’ experience working in KT and in managerial/supervisory roles, and followed a small group, self-directed, problem-based approach to learning. Both received training from the primary investigator on content such as appraisal and synthesis of evidence, implementation strategies, and small group learning approaches. While specific activities varied across the cases, generally the intervention targeted six of seven steps in the evidence-informed public health process defined by the National Collaborating Centre for Methods and Tools (NCCMT) [30]. This included defining a practice-based question; searching effectively and efficiently for research evidence; appraising the scientific merit of research evidence; synthesizing the evidence; assessing the applicability and transferability of the evidence to the local setting; and implementing changes in programs and practices. Examples of changes to programs that could have occurred include: starting a new program, adding new activities to an existing program, removing some activities from an existing program, or stopping a program altogether. Given the cases identified important issues for their context at the time of the study, changes to practice could vary from case to case and could cover the full continuum of public health programs and services (i.e., chronic disease prevention to communicable disease control to environmental health). The KT intervention included large and small group workshops that included both didactic and interactive components; face-to-face, electronic, and telephone communication between health department staff and the KBs; and meetings and presentations with senior management by the primary investigator.

Each case was encouraged to tailor the intervention to suit their particular needs at baseline and throughout the intervention. This resulted in differences across cases with respect to intervention implementation. It also ensured that the intervention remained appropriate and relevant to the case’s needs as organizational changes occurred. Case A, which was already actively engaged in promoting EIDM at baseline, requested the KB be physically onsite 2 days per week and provided a designated work area. Case B originally had the KB physically onsite 2 days per month, but this evolved over time to become half a day every week onsite, meeting with staff in the various offices in which they worked across the region. Case C also started with the KB onsite 2 days per month, but changed to 1 day every other week, with a designated work space. For the latter two cases, when the KB was not onsite, ongoing intervention occurred via email and telephone. A more detailed description of the intervention can be requested from the primary author [31].

### Data collection

Data were collected to explore characteristics related to the cases, participants, and the context in which the cases functioned. Data collection techniques included an organizational self-assessment conducted via focus groups, online surveys, and an in-person skills assessment. Data were collected at baseline and immediately post intervention (22 months after the intervention began). For data collection and analysis purposes, all participants were classified as belonging to one of three groups, based on the extent of their involvement in the intervention: 1) not involved in the intervention; 2) involved in large-group training (e.g. department-wide workshops and/or presentations), but not directly involved with the KB; and 3) intensively involved (i.e. as members of project teams or with one-on-one mentoring with a KB). A description of each data collection method follows.

#### Organizational self-assessment

Data related to organizational capacity to acquire, assess, adapt and apply research evidence in practice was collected using a validated organizational self-assessment tool developed by the Canadian Foundation for Healthcare Improvement, called “Is Research Working for You” [32, 33]. The tool consists of 40 statements and respondents individually rate the extent to which each statement reflects the organization using a 5 point scale (1 = strong disagree, 2 = disagree, 3 = neither agree or disagree, 4 = agree, 5 = strongly agree). The KB administered the assessment via focus groups with project-specific teams at each case at the beginning of the intervention. The intent of the focus group discussion was to reach a consensus rating for each statement. These discussions enabled the research team to gather a more in-depth understanding of each organization at baseline and informed further tailoring of the KT intervention.

#### EIDM knowledge and skills

To assess knowledge and skills related to EIDM, an in-person skills assessment was administered to participants involved in the KT intervention. The EIDM Skills Tool was developed to address a gap in the literature on evaluating knowledge and skills for EIDM [34]. The tool consists of 18 scenario-based questions in both short answer and multiple choice formats. Participants were asked to: formulate a question using the PICO framework (i.e. Population, Intervention, Comparison, Outcome); indicate where they would search for research evidence and evaluate the rigour of a given search strategy; critically appraise single studies and systematic reviews; interpret the results of a forest plot; and apply research evidence to practice scenarios. This tool was administered in 30-min in-person sessions at baseline and again immediately post intervention. Scoring of the tool was done with a marking key and consisted of summing responses to the items for a total score that ranged from 0 to 36. Three members of the research team (RT, KD, JY) independently graded five completed EIDM Skills Tools and compared their results, discussing any discrepancies to reach consensus. The remaining tools were graded by one member of the research team (RT).

#### EIDM Behaviours

An online survey was used to gather data on demographic information, self-reported EIDM behaviours, and how knowledge “flows” within the organization via social networks. The tool is available upon request from the primary author. The survey, which took approximately 20 min to complete, was administered to all staff working within service departments at each case, including those not directly involved with the intervention, at baseline, and follow-up. This strategy resulted in a different number of staff participating in baseline data collection from those at follow-up. This was as a result of two factors: 1) the number of staff employed at a case increased during the study period; and/or 2) increased knowledge about EIDM as well as a greater expectation to participate in the study at follow-up may have resulted in more staff completing the survey than at baseline. Staff received an email invitation from senior management with a link to the survey. A unique identification code assigned to each participant allowed the data to be linked longitudinally.

The first section of the survey consisted of seven demographic-focused questions. All questions had to be completed in order to proceed to the next section. Participants were asked about: sex; number of years employed in public health; highest degree earned; professional designation (if applicable); directorate/division in which they worked; job title; and previous experience with EIDM.

The second section of the survey included the Evidence-Based Practice (EBP) Implementation in Public Health Scale [35]. The EBP Implementation scale, consisting of 18 questions, represents a systematic approach to measuring engagement in EIDM-related behaviours, such as: searching for and appraising evidence; sharing evidence among colleagues; and using evidence in practice. This scale has been shown to have good reliability and validity [35]. Participants were asked to read each statement and circle the number that best described how often (e.g. 0 times, 1–3 times, etc.) the statement applied to their practice in the past 8 weeks. The option of “Not applicable to my role” was added to each statement after consultation with the tool developers. Participants were required to complete all 18 questions before proceeding. The scale is scored by summing the responses for the 18 items (possible total score of 0 to 72); a higher score indicates greater frequency of EIDM behaviours [35].

### Data analysis

For the organizational self-assessment where more than one team completed the assessment, an average rating across teams was calculated for each statement for each case. Ratings across the three cases were categorized into two groupings: those that were 4 and higher as indicative of items the cases were strong in; and less than 2.5, indicative of an item the case was weak in. The average ratings across cases were calculated by a member of the research team (RT) and organized in a table, identifying overall trends across the cases with respect to EIDM.

The sum scores of the EIDM Skills Tool (knowledge and skills) and EBP Implementation Scale in Public Health (behaviour) were calculated for each participant. Participant scores from each case were aggregated to represent an organizational value for each organization for each outcome. Mixed effects regression analyses were used to calculate the marginal means of EIDM knowledge and skills scores and behaviour over time. The fixed effect variables were the interaction between the level of involvement in the intervention (intensively involved vs. the other two groups) and time (baseline, follow-up), to assess the effect of involvement on the longitudinal changes of scores. Two level random effects variables were individual participants nested within the three cases. Marginal means of EIDM knowledge and skills scores and behaviour were calculated and reported by involvement level at each time point. Since the regression models did not include any covariates, the marginal means for each level of involvement in the intervention at either time point represented the average scores of the dependent variables for a notional individual at the center of the distribution of random effects and were used for ease of interpretation [36]. All analyses were carried out in STATA 13.1.

## Results

### Baseline characteristics of the sample

#### Cases

The three cases varied considerably with respect to organizational structure, populations served, and priority of EIDM within the organization (Table 1).

**Table 1:** General description of health departments and the regions they serve

	Case A	Case B	Case C
Population	1,200,000	2,700,000	760,000
Area	1254 km^2^	630 km^2^	2590 km^2^
General notes on population served	Large, diverse immigrant population	Large, diverse immigrant population	Mid-size, mix urban/rural area served
	Large municipality in Canada	Large municipality in Canada	
	High rate of annual population growth	Large urban centre	
Total Workforce	620	1750	741
Organizational Structure (Divisions / Directorates)	Office of the Medical Officer of Health	Communicable Disease Control	Emergency Medical Services
		Chronic Disease and Injury	Environmental Health
	Chronic Disease and Injury Prevention	Prevention	Oral Health
		Healthy Communities	Public Health Nursing and Nutrition
	Environmental Health	Healthy Families	Infant and Child Development Services ^a^
	Family Health	Healthy Public Policy	
	Communicable Disease Control	Performance and Standards	Administration ^a^
		Healthy Environments ^a^	
		Strategic Support ^a^	
		Dental and Oral Health ^a^	
		Finance and Administration ^a^	
Office Location(s)	One central office; three satellite offices	One head office; 14 additional office locations (and 19 clinics, 1 warehouse)	One central office
Strategic Plan / Infrastructure Priorities	Developing the workforce	Deliver services that meet the health needs of a diverse communities	Explore new media to deliver health messages.
	Making evidence-informed decisions		
	Measuring performance		Understand, promote and advocate for the social determinants of health.
	Enhancing external / internal communications	Champion healthy public policy	
		Plan for and respond to urgent public health threats and emergencies	Understand and improve outreach to priority populations through improved service accessibility and effective social marketing.
	Serving an ethno-culturally diverse community		
		Lead innovation in urban public health practice	
	(Strategic Plan, 2009–2019)		
			Advance research and knowledge exchange through interdivisional collaboration and innovation.
		Be a healthy workplace that embraces excellence and promotes collaboration and mutual respect	
			(Quality Enhancement Plan, 2008–2010)
		(Strategic Plan, 2010–2014)	

^a^Directorates not included in PHSI study

This information represents the health departments at baseline (2009–2010)

#### Case A

Case A serves a very large, mostly urban population within a moderately large geographical area. It served a diverse immigrant population and had been experiencing a high rate of annual population growth, particularly among immigrants for a number of years. There were a total of 620 public health professionals working within five divisions from primarily one central location. Staff from all divisions were encouraged to participate in the study and the number of eligible staff remained constant throughout the study. At baseline, EIDM was identified as one of nine strategic priorities within the organization’s 2009–2019 strategic plan.

#### Case B

Case B serves a large, mostly urban population distributed in the smallest geographical area within a very large urban centre. More than 1700 public health professionals from nine directorates worked in this organization who were dispersed among one head office and 14 additional offices. Staff from five directorates - those most directly involved in service provision to the public - were encouraged to participate in the study. The number of eligible staff did not change substantially during the study. At baseline, Case B did not have a strategic priority directly related to EIDM.

#### Case C

Case C serves an urban/rural population distributed in a broad geographical area. It too had experienced a high rate of annual population growth over several years, which resulted in significant increases in the number of full time equivalent staff employed by the organization both prior to the study as well as during the study. There were 741 staff working in six divisions, predominantly in one central office. Staff from the five divisions most directly involved in service provision were encouraged to participate in the study, which at baseline was 201 public health professionals and 309 by follow-up. At baseline, EIDM was inferred as a strategic priority without directly using the term EIDM.

#### Individuals

A total of 606 public health professionals across the three cases responded to the baseline survey: 207 (33% of those invited to participate) from Case A, 304 (28%) from Case B, and 95 (47%) from Case C. The sample is described in greater detail in Table 2. In summary, almost all respondents were female with an average of 12 years working in public health. Approximately 56% held a baccalaureate degree and 32% a graduate degree. Most respondents worked in chronic disease prevention, sexual health and communicable diseases, family health, and environmental health, with the majority being a public health nurse, supervisor/manager, public health inspector, and health promotion consultant. Self-reported experience in EIDM ranged from 0 to 25.75 years at baseline. Among the 606 participants, 77 became very involved in the intervention, 158 somewhat involved, and 371 not at all involved. A larger proportion of participants in Case B held graduate degrees and had more years of experience working in public health compared to the other cases, both which were statistically significant (chi^2^ = 38.6, *p* < 0.001). Furthermore, the intensively involved staff from Case A had the highest average EIDM behaviour (F = 3, *p* < 0.05) and knowledge and skills scores (F = 4.2, *p* < 0.02) at baseline, compared to the other cases (Table 2).

**Table 2:** Baseline characteristics of study participants

		Case A		Case B		Case C	
		Intensively involved	Others	Intensively involved	Others	Intensively involved	Others
Female (%)		44 (92%)	143 (90%)	12 (100%)	266 (91%)	15 (88%)	61 (78%)
Years of public health experience; mean (SD)		12 (9)	10 (8)	14 (7)	14 (9)	11 (7)	11 (9)
Degree	Diploma	0	26 (16%)	0	28 (10%)	1	19 (24%)
	Baccalaureate	24 (50%)	103 (65%)	4 (33%)	144 (49%)	13 (76%)	49 (63%)
	Masters	22 (46%)	29 (18%)	8 (67%)	111 (38%)	3 (18%)	9 (12%)
	Doctorate	1	1	0	9 (3.1%)	0	1
EIDM behaviour		12 (7)	10 (9)	8 (7)	10 (10)	8 (5)	8 (7)
EIDM knowledge and skill		12 (6)		10 (3)		10 (2)	
Three most frequent professional roles (number)		Public health nurse (69), supervisor (29), health promotion officer (16), manager (16)		Public health nurse (109), consultant (73), manager (41)		Public health nurse (48), public health inspector (16), manager (12)	
Three most frequent Divisions (number)		Chronic Disease and Injury Prevention (57), family health (50), Communicable Disease Control (44)		Communicable Disease Control (83), Healthy Families (83), Healthy Living and Chronic Disease Prevention (60)		Public Health Nursing and Nutrition (55), Environmental Health (21), Administration (7), Emergency Medical Services (7)	

Among the staff who participated in the baseline assessment in Case A, 48 respondents became intensively involved in the intervention and 33 participated in large-group training. About 46% of the intensively involved staff held Masters or doctorate degrees, compared to 18% among those not intensively involved (chi^2^ = 25, p < 0.001). In Case B, 12 staff were intensively involved in the intervention and 76 participated in large-group training. These 12 did not differ significantly from the rest of the staff in Case B in terms of work experience or education, though more intensively involved individuals had Masters or doctorate degrees (67% vs 41%). In Case C, 17 respondents were intensively involved and 49 participated in large-group training. The intensively involved group did not differ significantly from the others in Case C. At follow-up there were a total of 804 participants, 258 (42%) from Case A and 391 (37%) and 155 (50%) from Case B and C, respectively. The impact of more staff participating in the EIDM behaviours survey at follow-up compared to baseline is difficult to estimate. Staff who became employed at the cases following baseline data collection may not have been sufficiently exposed to the KT intervention to receive much benefit thereby resulting in limited impact of the intervention on their EIDM behaviours. On the other hand, given there was more awareness of the study at follow-up than at baseline, more staff may have felt compelled to complete the follow-up survey to illustrate their favorable attitudes toward EIDM. This could have biased the results in the direction of showing a positive impact of the KT intervention. The expectation is that these opposing biases likely had minimal impact on the overall findings reported in this paper.

### Organizational Self-Assessment for EIDM

Common themes identified as strengths and/or weaknesses across the cases are presented in Table 3. Common across the three cases, at baseline, was the belief that using research evidence was a priority for the organization. Cases A and B indicated that management clearly communicated the organizations’ strategies and priorities; Case C reported a corporate culture that valued and rewarded flexibility, change and continuous quality improvement. In Case A, the Medical Officer of Health and Associate Medical Officer of Health were actively involved in efforts to embed EIDM within the organization and acted as internal champions for participation in this study. In Cases B and C, the role of internal champions was delegated to Directors, with the Medical Officers of Health being supportive of the KT intervention but less actively involved. In all three cases, it was reported that the organization was weak in assessing, adapting, and considering research evidence in decision making and did not have enough skilled staff, time, and resources to increase capacity for EIDM. Furthermore, Cases A and B indicated they could improve on communicating internally in a way that ensured information was exchanged across the entire organization. Case A noted that one of their strengths was having arrangements with external experts who offer critical appraisal skills and tools, while Cases B and C indicated they had limited arrangements with external experts who could assist them with EIDM.

**Table 3:** Results of Organizational Self-Assessment for EIDM

Case	Strong (> 4)	Weak (< 2.5)
Case A	• Staff have incentive to use research in decision making • Look for research in journals • Look for research in non-journal reports • We learn from peers through formal and informal networks to exchange ideas, experiences and best practices • We have arrangements with external experts who use critical appraisal skills and tools to assess methodology and evidence reliability • Using research is a priority in our organization • Our organization has committed resources to ensure research is accessed, adapted, and applied in decision making • Management has clearly communicated our strategy and priorities so that those creating or monitoring research know what is needed in support of our goals • Relevant on-staff researchers are made part of decision making discussions	• Our staff have enough time for research • Our organization has enough skilled staff with time, incentives, and resources who use research communication skills to present research results concisely and in accessible language • Our organization has enough skilled staff with time, incentives and resources who use research communication skills to synthesize in one document all relevant research • Our organization has enough skilled staff with time, incentives, and resources who use research communication skills to link research results to key issues facing our decision makers • We communicate internally in a way that ensures there is information exchanged across the entire organization
Case B	• Our staff has the resources to do research • Look for research in journals • Look for research in non-journal reports • Look for research in databases by subscription or internet access • We learn from peers through formal and informal networks to exchange ideas, experiences and best practices • Our staff can relate our research to our organization and point out similarities and differences • Using research is a priority in our organization • Our organization has committed resources to ensure research is accessed, adapted, and applied in decision making • Decision makers in our organization give formal consideration to recommendations from staff who have developed or identified high quality research	• Our staff have enough time for research • We have arrangements with external experts who search for research, monitor research, or do research for us • Staff in our organization have the critical appraisal skills and tools for evaluating the quality of methodology used in research • Staff in our organization have the critical appraisal skills to evaluate the reliability of specific research • We have arrangements with external experts who use critical appraisal skills and tools to assess methodology and evidence reliability • We have arrangements with external experts to identify the relevant similarities and differences between what we do and what the research says • Our organization has enough skilled staff with time, incentives, and resources who use research communication skills to present research results concisely and in accessible language • Our organization has enough skilled staff with time, incentives and resources who use research communication skills to synthesize in one document all relevant research • We have arrangements with external experts who use research communication skills to present research concisely and in accessible language • We have arrangements with external experts who use research communication skills to synthesize in one document all relevant research • We have arrangements with external experts who use research communication skills to link research to key issues facing our decision makers • We have arrangements with external experts who use research communication skills to provide recommended actions to our decision makers • We have committed resources to ensure research is accessed, adapted, and applied in making decisions • We communicate internally in a way that ensures there is information exchanged across the entire organization • Our corporate culture values and rewards flexibility, change, and continuous quality improvement • When we make major decisions we usually allow enough time to identify researchable questions and create, analyze, and consider research results and other evidence • Staff and appropriate stakeholders know when and how major decisions will be made • Staff who have provided evidence and analysis usually participate in decision making discussions • Relevant on-staff researchers are made part of decision making discussions • Staff and appropriate stakeholders receive feedback on decisions, with a rationale for the decision
Case C	• We learn from peers through formal and informal networks to exchange ideas, experiences and best practices • Using research is a priority in our organization • Our corporate culture values and rewards flexibility, change, and continuous quality improvement	• We look for information on websites such as Best Evidence • Staff in our organization have the critical appraisal skills and tools for evaluating the quality of methodology used in research • Staff in our organization have the critical appraisal skills to evaluate the reliability of specific research • We have arrangements with external experts who use critical appraisal skills and tools to assess methodology and evidence reliability • We have arrangements with external experts to identify the relevant similarities and differences between what we do and what the research says • Our organization has enough skilled staff with time, incentives, and resources who use research communication skills to present research results concisely and in accessible language • Our organization has enough skilled staff with time, incentives and resources who use research communication skills to synthesize in one document all relevant research • Our organization has enough skills staff with time, incentives, and resources who use research communication skills to link research results to key issues facing our decision makers

Teams from Case A indicated that staff in their organization had the incentive to do EIDM, with committed resources and staff roles to ensure research evidence is accessed, adapted, and applied in making decisions. Conversely, Case B identified this as one of their weaknesses and both Cases B and C reflected on a current lack of committed resources for staff to engage in EIDM. All three organizations indicated a weakness in *summarizing* research results in a user-friendly way, such as having staff skilled in research communication and with time, incentives, and resources available to present and synthesize research results concisely and in accessible language. Cases A and C reported that their organization was strong in learning from peers and exchanging ideas through informal and formal networks. In contrast to this, Cases A and B reflected that information exchange across the *entire organization* was limited.

### EIDM knowledge and skills

Knowledge and skills were assessed at baseline and follow-up among those involved in the intervention (with the KB or large group training). In Case A, the number of respondents was 44 at baseline and 40 at follow-up all of whom were intensively involved in the intervention. In Case B, 88 completed the assessment at baseline and 43 at follow-up, the majority of whom participated in large-group training. In Case C, 20 staff completed the assessment at baseline and 15 at follow-up, the majority of whom were intensively involved in the intervention.

Table 4 shows the marginal means (SE) of EIDM knowledge and skills scores at baseline and follow-up in each case and pooled across cases. A statistically significant improvement in knowledge and skills scores was observed from baseline to follow-up in all three cases, with an improvement from 11.9 to 16.5 in Case A, from 9.5 to 10.5 in Case B, and from 9.6 to 13 in Case C. Similarly, the pooled analysis of scores across all three cases showed a statistically significant increase from 10.6 at baseline to 13.4 at follow-up (Table 4).

**Table 4:** Marginal means (SE) of EIDM behaviour and knowledge and skills scores at baseline and follow-up

		Case A†	Case B†	Case C†	Pooled ‡
EIDM behaviour	Baseline	10.1 (0.5)	10.2 (0.5)	7.9 (0.7)	9.5 (0.6)
	Follow-up	10.6 (0.5)	10.5 (0.5)	8.0 (0.7)	9.8 (0.6)
EIDM knowledge and skill	Baseline	11.9 (0.8)	9.5 (0.4)	9.6 (0.7)	10.6 (1.0)
	Follow-up	16.5 (0.9)***	10.5 (0.5)*	13 (0.8)***	13.4 (1.0)***

*p < 0.05; ***p < 0.001

†Marginal means obtained from a mixed effects regression model including time, with individuals as random factors, separate for each health department. P values show the comparison between baseline and follow-up

‡Marginal means obtained from a mixed effects regression model including time, with individuals nested in health departments as random factors. *P* value shows the comparison between baseline and follow-up

### EIDM behaviour

Comparison of the EIDM behaviour scores from baseline to follow-up did not show any improvement in behaviour among participants in any case, as shown in Table 4. In Case A, the mean (SE) of EIDM behaviour scores was 10.1 (0.5) and 10.6 (0.5) at baseline and follow-up, respectively. In Case B, the mean (SE) of EIDM behaviour scores was 10.2 (0.5) at baseline and 10.5 (0.5) at follow-up. In Case C, it was 7.9 (0.7) and 8 (0.7).

When respondents were analyzed according to their level of involvement in the intervention, the staff who were intensively involved showed improvement in EIDM behaviour. Table 5 shows the marginal means (SE) of EIDM behaviour scores at each time point for the different involvement groups by case, obtained from separate mixed effect models for each case. At baseline, the intensively involved group had higher EIDM behaviour scores (mean (SE) of 12.7 (1.1)) than non-involved respondents (mean (SE) of 9.8 (0.6)) in Case A (*p* = 0.02). The group who was intensively involved in Case A also showed a statistically significant improvement in EIDM behaviour at follow-up from a mean score of 12.7 to 14.9 (*p* = 0.04). Similarly, in Cases B and C, the intensively involved groups improved from 9.7 and 9.1 to 10.4 and 10.5, respectively, although the changes did not reach statistical significance.

**Table 5:** Marginal means (SE) of EIDM behaviour scores for each involvement level in each health department†

	Case A		Case B		Case C		Overall	
	Baseline	Follow-up	Baseline	Follow-up	Baseline	Follow-up	Baseline	Follow-up
Not involved	9.8 (0.6)	9.4 (0.6)	10.3 (0.6)	10.5 (0.6)	5.3 (1.2)	4.4 (1)	9.1 (0.8)	9.0 (0.8)
Large-group training	8.2 (1.2)	9.7 (1.2)	10.2 (1.1)	10.5 (1.4)	9.4 (0.9)	10.2 (0.8)	9.5 (0.9)	10.4 (1.0)
Intensively involved	12.7 (1.1)	14.9 (1.2)*	9.7 (2.8)	10.4 (3.1)	9.1 (1.6)	10.5 (2.1)	11.4 (1.2)	13.2 (1.3)

*:*p* < 0.05

†Marginal means obtained from a mixed effects regression model including interaction between time and involvement, with individuals as random factor, separate for each health department, and overall. *P* values show the pairwise comparison of marginal means with baseline values in the same involvement group

The data of all three cases were pooled in a mixed effects regression model to predict the EIDM behaviour scores. The marginal means of scores at baseline and follow-up for the different involvement groups are reported in Table 5. The regression analysis showed an improvement in behaviour scores of the staff who were intensively involved with the intervention from 11.4(1.2) at baseline to 13.2(1.3) at follow-up, although not statistically significant (*p* = 0.06).

## Discussion

The purpose of this study was to evaluate the impact of a KT intervention delivered by KBs on EIDM knowledge, skills and behaviour among public health professionals working in three public health departments in Ontario, Canada. The three cases varied with respect to size of the population served, number of full time staff employed, education, years worked in public health, and existence of a strategic priority for EIDM. They also differed on how often the KB was physically onsite and the level and type of engagement of staff in the KT intervention. Furthermore, those participating in data collection represented three unique involvement groups: those intensively involved in the intervention, those who received some intervention usually in large group training sessions, and those who were not at all involved in the intervention.

The intervention was delivered by two KBs and focused on the development of EIDM knowledge, skills, and behaviour among public health professionals within each case, as well as the development of organizational structures and processes that supported EIDM. More specifically, the KBs mentored public health professionals one-on-one or in small teams through reviews of the evidence, conducted large group training workshops, and presented at meetings at all levels in the organization. In addition, the primary investigator (MD) met with senior management throughout the intervention to discuss study progress as well as strategize about development and implementation of organizational mechanisms to support EIDM. The activities implemented by the KBs in this study align well with tasks undertaken by KBs evaluated in other studies [25, 37] whose roles can generally can be categorized into knowledge managers, linkage agents, change agents, and capacity developers.

The results of this study indicate that knowledge brokering is an effective but context-dependent strategy for facilitating EIDM, as statistically significant improvements in knowledge and skills were observed in all cases and overall, and EIDM behaviour increased among those most intensively involved in the intervention, particularly in the health department with the greatest number of engaged participants. Generally, there is support in the literature for these findings with two systematic reviews reporting KBs as an effective KT strategy for facilitating evidence-informed practice [37, 38]. Another review however, concluded the effect of knowledge brokering on EIDM was unclear and more research was needed [25]. Still, others have reported increased knowledge and skills following exposure of public health professionals to a KB intervention similar to the one evaluated in this study [39].

The variation in findings may be related to factors such as where the KB delivered the intervention from and how the KB intervention was implemented. For example, Bornbaum et al., 2015 suggest that the physical location of the KB (i.e. internal rather than external to the organization) may play an important role in the impact of KT interventions [25]. In this study, while the KB was not a staff member of – and was therefore external to – the organizations, the KB delivered the intervention completely onsite in Case A and for the majority of the time in the other two cases. The largest magnitude of effect was observed in the case where the KB delivered all of the intervention onsite. It is likely that physical presence encourages face-to-face interaction, which in turn supports the development of trust and credibility as the KB builds relationships with staff. This is supported by others who have reported interpersonal contact with a KB is an essential condition for effective knowledge brokering interventions among health professionals [40, 41].

Furthermore, the results of this study indicate a key ingredient of the intervention was active engagement with the KB, either through one-on-one interaction or in small groups, over a prolonged period. Those who were actively involved with the KB reported significantly greater improvement in EIDM knowledge, skills, and behaviour, in comparison to those with limited involvement (generally through large group training workshops). This was also observed by Elueze, 2015 who found that studies in which participants were actively engaged with a KB tended to report positive effects on evidence-informed practice.

Another explanation for the positive results observed in this study may relate to each case designing the KT intervention they received as well as tailored throughout the intervention to suit their unique and specific needs. As noted earlier, each case determined how often the KB was onsite, what activities the KB conducted, and how staff would engage with the KB (one-on-one, small group, large group). Tailoring of the intervention to suit specific organizational needs has been reported by others to play an important role in improved EIDM. In a realist review of strategies to promote evidence-informed healthcare, McCormack et al., 2013 reported that context is a key factor in EIDM and that tailoring KT interventions to suit a particular context is likely to yield the greatest impact on evidence-informed practice [42].

Our findings lend ongoing support to the importance of organizational factors as facilitators and/or barriers of EIDM within an organization. Strategic priority, leadership, readiness, and choice of staff chosen to engage in training with the KB emerged from this study as important organizational factors with respect to implementation of the intervention and its observed impact. Similar results have been reported by others [37, 38, 42, 43].

### Strategic Priority

All three cases identified EIDM as important to its strategic goals but varied in the extent to which it was explicitly identified. For example, Case A explicitly identified EIDM as one of its strategic priorities in formal organizational documents at baseline; the organization was engaged in activities to realize this strategic priority prior to implementation of this study. Case B, at baseline, was sufficiently interested in EIDM to commit substantial resources to participate in this study but did not have a strategic priority directly related to EIDM in place. Senior management from Case C discussed EIDM as being important to the strategic goals of the health department but did not explicitly identify EIDM as a strategic priority, nor was the term EIDM specifically used. The findings of this study suggest the importance of identifying EIDM as a strategic goal at an organizational level. Given EIDM requires significant change in decision making processes, it may be that to affect this amount of change, it needs to be explicitly articulated as a priority, with a coinciding plan developed that outlines objectives, activities, expected outcomes and evaluation activities. Stetler et al., 2009 similarly reported that an organization that had designated EIDM as a strategic priority demonstrated greater gains in realizing this goal than an organization without a defined strategic goal [43]. Ways in which the strategic priority was identified in the organization included: verbal communication and recurrent EIDM language used within the organization; wording within key organizational documents such as vision and mission statements; and written role performance/expectation documents. Given movement toward EIDM generally requires significant change in how health departments function, a major organizational commitment, which is driven by identifying it as a strategic priority, may be required to provide adequate impetus for change.

### Leadership

Another key organizational component was leadership, with styles and levels of involvement in the study varying considerably across the organizations. These differences may have impacted how staff perceived organizational commitment to EIDM, as well as the expectation to participate in the KT intervention and to ultimately engage in EIDM behaviour. The case where the Medical and Associate Medical Officers of Health actively encouraged participation in the study, participated themselves in the intervention, and consistently encouraged EIDM behaviours throughout the organization had the greatest number of staff who became actively involved in the intervention and reported the greatest gains in EIDM knowledge, skills, and behaviour from baseline to follow-up. While improvements were also noted for EIDM outcomes in the other cases, fewer staff became actively involved in the KT intervention. It is possible that active involvement in the KT intervention among those in the most senior positions in the organization, is a critical component in making progress toward EIDM. It also highlights the importance of senior leaders in an organization delineating, at the outset of a KT intervention, what roles and responsibilities they will commit to as part of the initiative. Similar findings have been reported by Stetler et al., 2006, who found support by organizational leaders for EIDM was a key factor in implementation efforts within the Veterans Health Administration [44]. In another study to promote evidence-based practices in addiction health services, leadership strategies found to be associated with use of research evidence in practice included: demonstrating knowledge; proactively facilitating implementation; proactively creating a climate conducive to implementation; supporting change through individualized connections; and perseverance through problem solving [45]. Stetler et al., 2006 also noted that senior decision makers are the holders of an organization’s values and pivotal to the way in which an evidence culture is promoted and operationalized [44]. These findings demonstrate how crucial leadership is to EIDM: leaders must become actively engaged in EIDM to support others in the organization to follow suit. In planning KT strategies, organizations should consider starting with identification of the roles, responsibilities, and behaviours senior leaders will assume.

### Readiness

Presence of an explicit strategic priority for EIDM may have indicated a level of readiness for change that is essential for an organization and its staff to really engage in the change process. The three cases varied in the amount of previous efforts to facilitate EIDM, with Case A having been involved in a number of training and other EIDM initiatives, while the other two cases less so. Staff in Case A may have then been more familiar with EIDM and initially more interested in participating in the KT intervention than staff in the other cases. Their previous exposure to EIDM activities may have contributed to a greater level of excitement about EIDM and a greater understanding of what the term meant for specific individuals that promoted engagement by staff in the KT intervention.

Furthermore, as a result of previous EIDM initiatives, Case A further customized the KB component of the KT intervention to meet its specific needs and structure at the outset of the intervention. While the other cases made similar changes to the KB intervention, it took several months for Cases B and C to determine what best suited their unique needs and structures, which may explain differences in observed impact across the three cases. McCormack et al., 2013 also found readiness to be an important factor noting when there is compatibility between a change agent’s characteristics and approach and organizational conditions, then there is a greater chance the KB’s efforts will be successful [42].

Differences across cases in the KB intervention were also noted with respect to how the KB interacted with staff during the intervention. McCormack et al., 2013 found in their review evidence that integration of a ‘change agent’s’ role into an organization was a key factor in supporting EIDM [42]. In Case A, the majority of the KB’s time was spent supporting teams through reviews of the evidence, whereas in Cases B and C, a greater proportion of time was spent conducting large group EIDM workshops for staff, with a much smaller group involved in rapid evidence reviews. The results suggest that the amount of time spent in face-to-face interaction with a KB either one-on-one or in small groups, may be a critical component for KT interventions. In addition, the results also suggest that time spent actively engaged in EIDM activities, like those performed during the reviews of the evidence, that have direct relevance to one’s practice as well as the organization may be what is needed to produce significant changes in EIDM knowledge and skills. Similar findings were reported by Sarkies et al., 2017 whose systematic review evaluating the effectiveness of KT interventions to promote evidence-informed policies and management decisions in healthcare found that interactive strategies, including practice exercises that were of direct relevance to one’s organization and/or community, were positively associated with EIDM [46].

Cases B and C needed time in the early days of the intervention to develop a culture that was supportive of EIDM practice and ready to interact with the KB; case A had spent this time developing the culture prior to the start of this study. An organization, at some point, must invest the time in developing the culture, and it is likely that early initiatives encouraging EIDM can help ‘kick start’ this change.

All cases determined that having the KB onsite more regularly (weekly or biweekly, rather than monthly) helped maintain momentum in EIDM-related work and supported staff in prioritizing time allocated for EIDM. One knowledge brokering study from Scotland determined that timing played a key role in policy makers being ready to adopt evidence into decision making. The authors reported that the timing of disseminating research evidence converged with government priorities and initiatives which contributed greatly to policy makers’ receptivity to using the evidence in decisions [47].

### Choice of staff to receive training from the KB

The findings also indicate that who is chosen among staff to receive EIDM training can have an important effect on intervention impact. Generally, those who became actively engaged in the intervention exhibited greater EIDM behaviour at baseline and continued to increase their EIDM behaviours during the study. While the intervention effects were observed largely among those who were intensively involved in the intervention, other publications from this study illustrate that intensively involved staff, who were identified by their peers as central actors (i.e. people who are connected to many others and influence others) in the organization, influenced the EIDM behaviour of their peers, including those who were not involved in the intervention [48]. This suggests that a key factor organizations can use to identify staff for EIDM training is current behaviour as this likely indicates a level of interest and willingness to learn new skills that can be leveraged to demonstrate quick gains. An organization can use quick gains such as this to demonstrate to others in the organization that improvements in EIDM are attainable, therefore supporting a culture shift toward EIDM more quickly. These findings are supported by the Diffusion of Innovation theory, suggesting that innovations are first adopted by innovators and then early adopters [49]. If organizations can accurately identify staff who are likely to be innovators and/or early adopters, then training efforts are likely to be more effective and potentially have a spin off-effect for other staff. Future research should focus on the development of a tool that accurately identifies staff who demonstrate potential to become an EIDM innovator or early adopter and focus early training initiatives on this group.

We also studied, using social network analysis, if the effect of the intervention diffused through formal and informal social networks; the full results of which have been reported elsewhere. We found that individuals who sought EIDM-related advice and information from intensively involved opinion leaders (i.e. central actors in information seeking networks) significantly improved their EIDM behaviour over time, even when they did not participate in the intervention themselves [48]. Among this group, the ones who were also friends with the intensively involved opinion leaders showed significantly greater improvement [48]. These findings suggest that opinion leaders who were actively involved in the KT intervention were able to use their formal and informal social relations to diffuse the effect of training interventions among their colleagues. This finding is consistent with the literature on the diffusion of innovations through opinion leaders [50, 51]. It also highlights the importance of trust and informal workplace connections in the diffusion of EIDM behaviour.

Assuming the significant social influence in diffusion of EIDM in public health organizations, these findings suggest it may not be necessary to train all staff in an organization to change individual behaviour, but rather a train-the-trainer type of model may be effective, if those who are chosen for training are recognized by their colleagues as knowledgeable and trusted. More research is needed to understand how EIDM behaviour is situated in everyday workplace communications and how those communications could be leveraged to promote EIDM culture in public health settings. Currently, many public health departments indicate that the level of investment to effect noticeable change toward EIDM is too large and therefore represents a significant barrier to initiating change. However, the results of this study suggest the possibility of achieving EIDM with less investment through use of a train-the-trainer type of approach. This may make it feasible for more organizations to initiate change if they can accurately identify those staff who are most likely to exert the greatest influence on organizational change. Future studies should explore if this hypothesis is correct.

### Limitations

There are some limitations in this study that may have impacted the results. While there were hundreds of staff available to participate in the study, the number of staff who engaged in the KT intervention, either intensively with the KB or large group training, was relatively small. It may be that more preliminary activities needed to occur to promote EIDM prior to implementing the study so as to ensure greater participation.

The generalizability of the results may also be limited to public health professionals who are interested and engaged in EIDM rather than the public health workforce in general. For example, those who became actively engaged in the KT intervention exhibited more EIDM behaviours than those who did not engage in the intervention. As discussed earlier, organizations may realize greater organizational changes toward EIDM by focusing change efforts on those identified as innovators or early adopters.

While organizational support for the study was high the public health departments and staff still faced competing priorities, most notably the need to maintain service delivery. This created challenges with respect to the amount of attention that could be allocated to the EIDM change efforts over the course of the 22 month KT intervention. While the implemented KT intervention was longer and more intense than most reported in the literature, it may have been insufficient to have a meaningful impact, particularly given the size of at least one of the organizations. Consideration of the size of an organization, as well as where an organization is in the change process, can be used to develop a more appropriate length and intensity of KT interventions.

Finally, timing of measuring outcomes may have impacted the results of this study. While the longitudinal design of this study was a strength, it is likely that a longer period of time was required in order to see meaningful change in EIDM behaviour. Given organizational change is a slow process, it makes sense that cases that were early in their journey toward EIDM when this study began, while progressing toward EIDM, did not achieve an impact with sufficient magnitude to reach statistical significance. Future studies should include follow-up periods lasting between 2 and 5 years following completion of the intervention.

## Conclusions

The findings of this study suggest there is reason for optimism regarding the effectiveness of KBs in facilitating EIDM knowledge, skills, and behaviours among public health professionals. While work remains to be done to more clearly understand how to support public health departments in embedding EIDM, these findings shed light on important components of an organizational KT intervention. The findings also highlight that it may be possible to train only a portion of the workforce while relying on the diffusion of innovations to motivate others in the organization to change. This has the potential to motivate more public health departments to engage in efforts to embed EIDM within its decision making processes. This study calls for the development of a tool to accurately identify staff who are likely to become EIDM innovators and early adopters, as well as determine what steps an organization could and should take to ‘get ready’ for a change initiative. Future studies should determine if these recommendations do in fact lead to significant improvements in EIDM.

## Abbreviations

CIHR: Canadian Institutes of Health Research
EIDM: Evidence-informed decision making
KB: Knowledge broker
KT: Knowledge translation
NCCMT: National Collaborating Centre for Methods and Tools
PHSI: Partnerships for Health System Improvement
